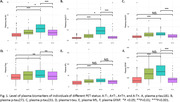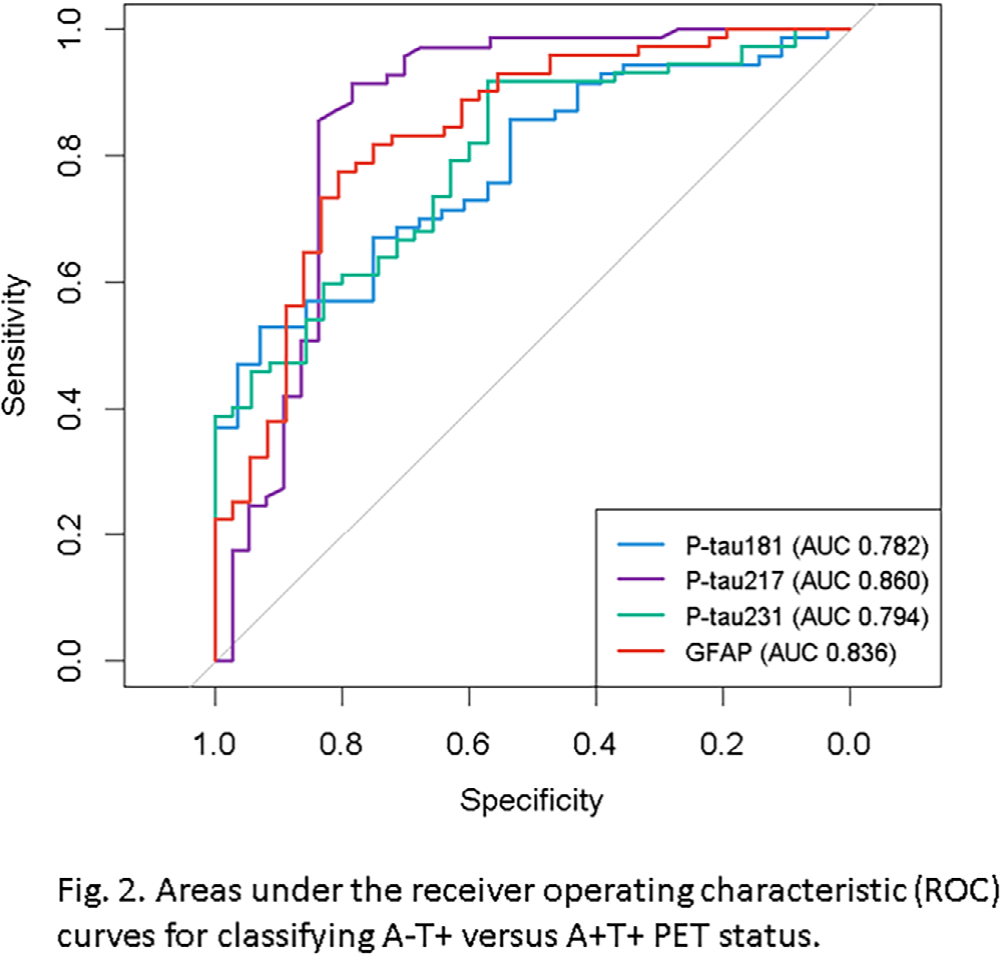# Characterization of plasma biomarkers of individuals with amyloid‐independent increased tau positron‐emission tomography uptake

**DOI:** 10.1002/alz.094118

**Published:** 2025-01-09

**Authors:** Tevy Chan, Joseph Therriault, Yi‐Ting Wang, Etienne Aumont, Jaime Fernandez Arias, Arthur C. Macedo, Nesrine Rahmouni, Stijn Servaes, Lydia Trudel, Seyyed Ali Hosseini, Yansheng Zheng, Kely Monica Quispialaya Socualaya, Sulantha Mathotaarachchi, Jenna Stevenson, Wan Lu Jia, Takashi Matsudaira, Brandon J Hall, Firoza Z Lussier, Serge Gauthier, Gallen Triana‐Baltzer, Hartmuth C. Kolb, Thomas K Karikari, Nicholas J. Ashton, Andrea L. Benedet, Henrik Zetterberg, Kaj Blennow, Tharick Ali Pascoal, Pedro Rosa‐Neto

**Affiliations:** ^1^ McGill University, Montreal, QC Canada; ^2^ Translational Neuroimaging Laboratory, The McGill University Research Centre for Studies in Aging, Montréal, QC Canada; ^3^ University of Pittsburgh, Pittsburgh, PA USA; ^4^ Department of Neurology and Neurosurgery, and Department of Psychiatry, McGill Centre for Studies in Aging, McGill University, Montreal, QC Canada; ^5^ Neuroscience Biomarkers, Janssen Research & Development, LLC, San Diego, CA USA; ^6^ University of Gothenburg, Mölndal, Gothenburg Sweden; ^7^ University of Gothenburg, Gothenburg Sweden; ^8^ Department of Psychiatry and Neurochemistry, Institute of Neuroscience and Physiology, The Sahlgrenska Academy, University of Gothenburg, Mölndal, Gothenburg Sweden

## Abstract

**Background:**

Increased uptake on Tau positron‐emission tomography (PET) is sometimes observed in the absence of amyloid ß accumulation. This A‐T+ PET profile might represent primary age‐related tauopathy (PART), an amyloid ß‐independent 3R/4R tauopathy observed in aging brains. Although A‐T+ individuals have been shown to follow a different cognitive trajectory compared to A‐T‐ and A+T+ individuals, it remains unknown how they differ in terms of plasma biomarkers. Here, we aim to characterize the plasma biomarkers of A‐T+ individuals.

**Method:**

This is a cross‐sectional study with data from the Translational Biomarkers in Aging and Dementia (TRIAD) cohort. Participants were classified into four categories based on their amyloid ß ([18F]AZD4694) and Tau ([18F]MK6240) PET status (A‐T‐, A+T‐, A+T+ and A‐T+). Plasma biomarkers phosphorylated tau (p‐tau)181, p‐tau217, p‐tau231, total tau (t‐tau), neurofilament light (NfL) and GFAP were compared using the Kruskal‐Wallis test with post hoc Benjamini‐Hochberg (BH) correction. Discriminative performance was assessed using the area under the receiver operating characteristic curve (AUROC).

**Result:**

Among the total 468 participants, 64 (13.7%) had A‐T+ PET status. The A‐T+ PET participants had a mean age 66.9 years (SD: 12.8), with 62.5% women and 39.1% showing cognitive impairment. Plasma p‐tau181, p‐tau217, p‐tau231 and GFAP were significantly higher in A+T+ individuals compared to A‐T+ (all p < 0.001). On the other hand, plasma p‐tau181 and 217 were significantly different between A‐T‐ vs A‐T+ (p<0.05), but not p‐tau231 and plasma GFAP. No significant differences were found in t‐tau or NfL regarding A‐T‐. Plasma p‐tau217 and plasma GFAP had the highest discriminative accuracies for A‐T+ vs A+T+ (AUROC: 0.860 and 0.836 respectively).

**Conclusion:**

Our data suggest that individuals with an A‐T+ PET status exhibit a different plasma biomarker profile than those with A+T+ and A‐T‐ status. Further characterization of fluid biomarkers could help identify this group of individuals and facilitate the differential diagnosis of adults with cognitive impairment. Furthermore, our results lend support to the use of plasma biomarkers for identifying amyloid ß PET positivity.